# Insect Larvae as an Alternate Protein Source in Poultry Feed Improve the Performance and Meat Quality of Broilers

**DOI:** 10.3390/ani14142053

**Published:** 2024-07-12

**Authors:** Muhammad Sajjad, Asif Sajjad, Ghazanfar Ali Chishti, Ehsaan Ullah Khan, Raimondas Mozūraitis, Muhammad Binyameen

**Affiliations:** 1 Department of Entomology, Faculty of Agricultural Sciences and Technology, Bahauddin Zakariya University, Multan 60800, Pakistan; sajjad8676@gmail.com; 2 Department of Entomology, Faculty of Agriculture and Environment, Islamia University, Bahawalpur 63100, Pakistan; asif.sajjad@iub.edu.pk; 3 Department of Animal Nutrition, Faculty of Animal Production and Technology, University of Veterinary and Animal Sciences, Lahore 54000, Pakistan; ghazanfar.ali@uvas.edu.pk (G.A.C.); ehsaan@uvas.edu.pk (E.U.K.); 4 Laboratory of Chemical and Behavioural Ecology, Institute of Ecology, Nature Research Centre, Akademijos Str. 2, 08412 Vilnius, Lithuania; 5 Department of Zoology, Stockholm University, Svante Arrhenius väg 18B, 10691 Stockholm, Sweden

**Keywords:** broiler performances, *Hermetia illucens*, meat quality, soybean replacement, *Spodoptera frugiperda*

## Abstract

**Simple Summary:**

Soybean meal is the main constituent of poultry feed, and the global supply of soybeans has decreased because of an imbalance between its demand and supply, primarily caused by the growing world population and reduced cultivation area. Therefore, a cheap and environmentally sustainable alternative protein source is urgently needed to maintain the sustainability of poultry production. In this study, we demonstrated that substituting 12% of soybean meal with either fall armyworm larvae or black soldier fly larvae enhances the growth, blood haematology, gut morphometry, and meat quality of broiler chickens. Consequently, soybean meal can be substituted with protein-rich insects in the poultry feed.

**Abstract:**

The primary challenge facing the global animal industry is the scarcity of protein feed resources. Various insects are gaining prominence as innovative feed sources due to their economic, environmentally friendly, and nutritious attributes. The purpose of the present study was to determine the effects of a partial replacement of soybean meal with fall armyworm *Spodoptera frugiperda* (Lepidoptera: Noctuidae) and black soldier fly *Hermetia illucens* (Diptera: Stratiomyidae) on the growth performances, blood parameters, gut histology, and meat quality of broilers. A total of 350 1-day-old (40 ± 0.15 g) male chicks (Ross 308) were randomly assigned to seven experimental meals. Each treatment was repeated five times with 50 birds per dietary treatment. The seven dietary treatments included 4, 8, and 12% replacements of SBM with larvae of *S. frugiperda* and *H. illucens*. SBM was the basal diet considered the control. The data showed that broilers fed 12% *S. frugiperda* or *H. illucens* exhibited a significantly higher (*p* < 0.05) live weight, average daily weight gain, and improved the feed conversion ratio. Meals with 12% *S. frugiperda* or *H. illucens* significantly enhanced (*p* < 0.05) haematological and gut histological parameters, including villus height, crypt depth, villus width, and villus height/crypt depth ratios. The meat of broilers fed the 12% *S. frugiperda* diet showed significantly higher (*p* < 0.05) lightness and yellowness. Replacing soybean meal up to 12% with either *S. frugiperda* or *H. illucens* larvae improves the growth performance, blood haematology, gut morphometry, and meat quality traits of broilers.

## 1. Introduction

Poultry meat and eggs are the best sources of quality protein and are needed by the millions of people living in poverty [1]. There will be around 9 billion people on Earth in 2050, which will result in an increased demand for food of animal origin [2,3,4]. Soybean meal (SBM) is the main constituent of poultry feed due to its superior quality protein contents, amino acid compositions, and ease of availability [5,6]. It provides essential amino acids (AAs), helping animals grow rapidly within a brief timeframe [7]. However, in Asia, the pervasive consumption of soybeans by humans, coupled with limited soybean production due to inadequate agricultural facilities and a shortage of suitable crops, leads to a deficiency of SBM for livestock and poultry feed [8]. Furthermore, its production is linked to several environmental disruptions, such as deforestation, soil erosion, eutrophication, use of excessive pesticides, biodiversity losses, and high CO_2_ emissions [9]. Mainstream costs in the poultry industry are related to feed; therefore, an alternative protein source with comparable nutritional value is urgently needed to ensure the long-term viability of poultry production [10,11]. Therefore, insects are the most substantial nutritional-rich protein source that may substitute vegetable protein in poultry feed [12,13]. 

Insects have attained great attention as prospective constituents of poultry feed due to their high protein contents, fats, vitamins, and minerals [14,15], higher feed conversion ratio than livestock [16], low space requirement, and great acceptance by poultry, fish, and omnivores [17]. Moreover, insects can play a decisive role as a sustainable feed source for poultry by efficiently transforming and valorising the bio-wastes into high-quality feed, thereby exerting minimal pressure on land, water, and energy resources [18,19]. Furthermore, the exoskeletons of insects are rich in chitin polysaccharides that enhance the palatability of poultry feed [20]. Moreover, insect meals contain chitin, chitosan, lauric acid, and antimicrobial peptides, which promote chicken health [21,22]. Chitin bioactive compounds can have antimicrobial and immunostimulant effects, making them a viable alternative to antibiotics and beneficial to gut health [23]. 

Several insect species, such as *H. illucens* and *Tenebrio molitor* (Coleoptera: Tenebrionidae), could serve as valuable feed sources in place of SBM because they are high in protein (37 to 63%) and have a better AA profile [12,24]. Moreover, insects possess a higher lipid content (15 to 49%), which can be extracted to produce biodiesel and the remaining defatted meal as a protein-rich source suitable for the feed industry [25]. The modifications in the origins and amounts of dietary protein have been observed to impact the structure of the intestines, the digestion and uptake of nutrients, and the overall growth of broilers [26,27]. The growth performance, blood haematology, gut morphology, and meat quality of broilers [28], Japanese quails [29], and barbary partridges [30] have been improved by *H. illucens* and *T. molitor* meals [31,32]. Hwangbo et al. [33] reported that the growth performances of the broilers improved when fed 10–15% *Musca domestica* diet; similarly, Okah and Onwujiariri [34] found a higher weight of broilers fed at 20 and 30% *M. domestica* meals. Altmann et al. [35] reported that the carcasses of Ross 308 male birds fed 15% BSF meals after 35 days were heavier than those fed SBM meals. A full-fat *T. molitor* meal may also reduce *Bacteroides–Prevotella* cluster and *Clostridium perfringens* in broiler chicks [28]. 

The poultry industry largely relies on SBM with the miner fraction of insect meals [36]. Abandoning soybeans and adopting insects is a healthier and balanced approach to poultry nutrition [13,17]. Therefore, using native insect species as an alternative protein source is a wise decision [37]. The *Spodoptera frugiperda* (Lepidoptera: Noctuidae) caterpillars feed on various crops. Full-grown larvae of Lepidopteran and Dipteran are frequently endorsed as feed ingredients due to their ease of cultivation and harvesting, substantial size, and rich nutrient composition [38,39]. Therefore, exploring the native insect species in the poultry industry is crucial for sustaining poultry production. To our knowledge, *S. frugiperda* is being used for the first time as a replacement for SBM in poultry feed.

This study aimed to assess the effects of graded levels of SBM replacement with the native insect species *S. frugiperda* on growth performance, blood haematology, intestinal morphometry, and meat quality in broiler chickens. For comparison, we also maintained similar replacement levels of exotic *H. illucens*. 

## 2. Materials and Methods

### 2.1. Institutional Review Board Statement

The experiments were conducted for 35 days in a controlled environment, a floor-rearing broiler house, in A block, UVAS, Ravi Campus, Pattoki. All the procedures were organised according to the guidelines and approved by the Ethical Review Committee (No. DR/495), University of Veterinary and Animal Sciences (UVAS), Lahore, Pakistan.

### 2.2. Insects 

Fall armyworm *Spodoptera frugiperda* was cultured on different host plants while black soldier flies *Hermetia illucens* were reared on different bio-wastes–poultry waste, household, grains, fruit, and vegetable under the laboratory conditions (25 °C ± 1 °C, R.H 65% ± 5%, and 16L: 8D) at the Department of Entomology, Faculty of Agriculture and Environment, The Islamia University of Bahawalpur. The adults of *S. frugiperda* and *H. illucens* were fed a 10% honey solution. Full-grown larvae were harvested, ground using a blender, frozen for storage, and then oven-dried at 70 °C for 24 h. Nutrient compositions, energy contents, and amino acid profiles of *H. illucens* (BSF) and *S. frugiperda* (% on DM Basis) larvae were analysed using proximate analysis, bomb calorimetry, and Biochrome^®^ amino acid analyser (Holliston, MA, USA) following standard techniques in the laboratory of the Department of Animal Nutrition at UVAS, Ravi Campus Pattoki (Table 1). The samples were analysed in triplicate.

### 2.3. Feeding Trial

The experiments were performed on male broiler chickens (Ross 308). The effects of replacing SBM at 4, 8, and 12% levels with S. *frugiperda* and *H. illucens* were studied on growth performances, haematology, gut morphometry, and meat quality of broilers compared to the control diet. Three hundred fifty 1-day-old chicks (average 40 ± 0.15 g) were randomly assigned to seven experimental meals (5 pens/treatment and 10 bird/pen). The shed was cleaned and disinfected before the birds’ arrival, and each group was placed in a clean floor pen, bedded with 3–4 inches of rice husk, with equal floor space, feeder, drinker, and heating lamp. The litter was covered with paper to prevent eating it during the first week. The birds had ad libitum access to feed, water, and light. The shed temperature was 35 °C for the first week, then 28 ± 2 °C until the end of the biological trials, with a relative humidity of 50 ± 5%. Infrared lamps maintained a stable temperature during the first three weeks. The photoperiod (18 h L: 6 h D) was maintained through artificial light. Chicks were vaccinated against different infectious diseases as per the protocol recommended by the local veterinarian. The pens were monitored daily to observe the birds’ clinical symptoms of diseases and mortality. 

### 2.4. Diet Formulation

Meals were formulated according to the standards of Ross [40]. The SBM was replaced with *S. frugiperda* and *H. illucens* in gradually increasing levels—4, 8, and 12%—referred to as SF4, SF8, and SF12 and HI4, HI8, and HI12, respectively. The meals were formulated according to three phases of rearing for each treatment (as recommended by Ross 308 guidelines): a starter (1–10 d), a grower (11–24 d), and a finisher (25–35 d). All the meals were isocaloric and isonitrogenous for each phase. Table 2, Table 3 and Table 4 present the ingredients of dietary treatments (starter, grower, and finisher) and their calculated and analysed compositions. 

### 2.5. Growth Performances 

Growth performances of the birds in terms of live weight (LW), average daily gain (ADG), daily feed intake (DFI), and feed conversion ratio (FCR) were determined during the experimental period (35 days). The LW of the birds was individually recorded from the beginning of the trial after 10, 24, and 35 d. The ADG and DFI were performed at individual and pen levels after each growth period, respectively, while the FCR was assessed for each growth period and the entire experimental duration. A high-precision electronic scale was used for all calculations. 

### 2.6. Haematological and Serum Parameters

At 35 d, two birds per pen were killed within a few hours after arrival by a competently trained person using the rapid decapitation method. Two and a half millilitres of blood were collected from the slain birds in EDTA and serum-separating tubes (IVEN Pharmatech Engineering Co., Ltd., Shanghai, China). A blood smear was prepared to evaluate the complete blood count (CBC) using one glass slide with a drop of blood without an anticoagulant from each bird [43]. The serum-separating tubes were allowed to clot upright at room temperature for two hours. The serum was isolated through centrifugation (centrifuge D-37520, Sigma Aldrich Chemie Gmbh, Darmstadt, Germany) at 700× *g* for 15 min and subsequently subjected to analysis for creatinine, glucose, cholesterol, total protein (T. protein), albumin (Alb), globulin (Glob) and uric acid. All these tests were performed at the University Diagnostic Lab, UVAS, Lahore, Pakistan. 

### 2.7. Gut Histology

The small tissues from jejunum (JE) and ileum (IL) of a small intestine were excised at slaughtering. Sections of 2 to 3 mm in length were extracted from JE and IL of each sampled bird, promptly rinsed with normal saline, and then preserved in a 10% formalin (BDH Middle East, Dubai, United Arab Emirates) solution for a minimum of 72 h. The 5 μm cut sections were placed on slides and stained with Lilee Meyer haematoxylin (Sigma Aldrich Chemie Gmbh, Darmstadt, Germany) and counter-stained with Eosin Y (Sigma Aldrich Chemie Gmbh, Darmstadt, Germany). After staining, the transverse sections were visualised using an Olympus CX23 light microscope (Olympus, Tokyo, Japan), and the images were examined by ImageJ software, IJ 1.46r (National Institute of Health, Bethesda, MD, USA) [44]. Twenty well-developed villi and crypts were used to measure the villus height (Vh), villus width (Vw), crypts depth (Cd), and Vh/Cd. The intestinal indices were studied at the Department of Anatomy and Histology, UVAS, Lahore.

### 2.8. Meat Quality

The effect of insect meals on meat quality parameters, including drip and cooking losses, meat pH, shear force, and meat colour, were evaluated from both Rt. and Lt. breast muscles—*pectoralis major*—of the broiler at the Department of Meat Science and Technology, UVAS, Lahore. These parameters were studied after 24 h post slaughtering. One breast section of the slain bird was used to evaluate the drip loss and the other for cooking loss. Meat samples, after weighing, were enclosed in plastic bags and suspended at 4 °C in refrigerated storage as recommended by [45] for drip loss. Moreover, cooking loss was determined by placing breast samples in plastic bags and subjected to cooking in a water bath at 82–85 °C until the core temperature of the meat samples reached 72 °C [46]. pH meter 3210 SET 2 (WTW Xylem Analytics, Weilheim, Germany) was used to assess the pH was estimated from three different places of the breast portion. CR-410 colourimeter (Konica Minolta INC, Tokyo, Japan) was used for estimating the meat colour, such as L* (lightness), a* (redness), and b* (yellowness) [47]. The cooked breast portion was kept in a polystyrene tray and cooled in a chiller at 04 °C to calculate the shear force. The meat samples were cut parallel to muscle fibres in a rectangular shape (1h × 1w × 2L cm) with a scalpel handle blade. Finally, Warner–Bratzler shear force (N/cm2) was assessed using a V slot blade with the help of a TAXT plus 100C texture analyser (Stable Micro Systems, Surrey, UK).

### 2.9. Statistical Analysis

The statistical analysis was performed using the SPSS software package (version 21 for Windows, SPSS Inc., Chicago, IL, USA). The pens were the experimental units used for evaluating growth performances (*n* = 5) while individual birds were used to determine haematology (*n* = 10), gut histology (*n* = 10) and meat quality traits (*n* = 10). The data were subjected to a completely randomised design using a one-way ANOVA followed by the Duncan Multiple Range test. Orthogonal polynomial contrasts were used for data analysis to check the linear and quadratic responses to increasing levels of *S. frugiperda* and *H. illucens* in the meals. General Linear Model (GLM) was used to test the effects of meals, intestinal sites, and their interactions on the intestinal morphometric indices using Repeated Measure ANOVA followed by Tukey’s test at alpha *p* ≤ 0.05. The meals, intestinal sites, and their interactions were the fixed factors. 

## 3. Results

### 3.1. Growth Performances 

The growth performances of broilers in terms of LW, ADG, DFI, and FCR are presented in Table 5. The initial LW of DOC chicks did not differ (*p* > 0.05) for all the experimental meals. The LW and ADG of broilers showed a highly significant difference (*p* < 0.001) among all the meals at 10, 24, and 35 d of age. At 10 and 35 d of age, the highest LW was recorded in the broilers fed with the 12% *H. illucens* and *S. frugiperda* meals, while the lowest LW was observed using the control diet. The LW was the highest in the broilers fed with 12% of *H. illucens* and the lowest in the control group at 24 d of age. The meals of *H. illucens* affected LW increase in a linear matter (*p* < 0.001) in the starter and finisher phases while linear and quadratic responses were registered (*p* < 0.001 and *p* = 0.007) in the grower phase. Similarly, *S. frugiperda* meals exhibited linear and quadratic effects on LW of broilers (*p* < 0.001 and *p* < 0.001).

The highest and the lowest ADG values were recorded in the broilers fed with 12% *H. illucens* and the control diet, respectively, across the feeding periods. The orthogonal polynomials contrasts showed that ADG increased linearly during d 1–10 (*p* = 0.001) and d 25–35 (*p* < 0.001), while linear and quadratic responses were determined during d 11–24 (*p* < 0.001 and *p =* 0.004) and d 1–35 (*p* < 0.001 and *p* < 0.001) feeding with *H. illucens* meals. Similarly, there were linear and quadratic responses during d 11–24 (*p* < 0.001 and *p* = 0.001), d 25–35 (*p* = 0.001 and *p* < 0.001) and d 1–35 (*p* < 0.001 and *p* = 0.02) feeding with *S. frugiperda* meals. 

The DFI did not differ significantly (*p* > 0.05) during the feeding periods. The FCR was significant (*p* < 0.05) among the treatments across the experimental periods. The highest FCR values were registered when broilers were fed the control diet, while the lowest values were recorded when the 12% *H. illucens* and *S. frugiperda* meals were applied. There was a linear response in FCR during d 1–10 (*p* = 0.002), d 11–24 (*p* < 0.001) and d 25–35 (*p* < 0.001), while linear and quadratic responses during d 1–35 (*p* = 0.003 and *p* = 0.04) in respect to *H. illucens* meals. The *S. frugiperda* meals affected FCR in a linear and quadratic matter during d 11–24 (*p* < 0.001 and *p* = 0.03), d 25–35 (*p* = 0.024 and *p* < 0.001) and d 1–35 (*p* = 0.045 and *p* = 0.02) while a linear response was determined during d 1–10 (*p* = 0.05).

### 3.2. Haematology

#### 3.2.1. Complete Blood Count of Broiler 

The complete blood count of the birds fed on different dietary treatments is presented in Table 6. These parameters differed significantly (*p* < 0.001) except for monocytes and EO (*p* > 0.05) among all the meals. The Hb, platelets, and total leucocyte counts were the highest in the broilers fed the 12% *S. frugiperda* diet and the lowest fed the 4% *H. illucens* diet. The highest RBCs, heterophils, and lymphocyte values were recorded in the broilers fed the 12% *S. frugiperda* diet and the lowest values were recorded when the control diet was used. The highest and the lowest values of HCT and MCV were observed when broilers were fed the 12% *S. frugiperda* and the 4% *S. frugiperda* diets. The highest MCH was registered in the broilers of the control group and the lowest MCH values were determined in the broilers fed the 8% *S. frugiperda* meal. The highest and the lowest MCHC values were determined in the broilers fed the 12% *H. illucens* and the 8% *S. frugiperda* diet, respectively. There were linear and quadratic responses in HCT (*p* = 0.031 and *p* < 0.001), MCH (*p* < 0.001 and *p* < 0.001), MCHC (*p* < 0.001 and *p* < 0.001), platelets (*p* < 0.001 and *p* < 0.001), total leukocyte counts (*p* < 0.001 and *p* < 0.001), heterocyst (*p* < 0.001 and *p* < 0.001), and lymphocyte (*p* < 0.001 and *p* < 0.001) to the *H. illucens* meals while a quadratic response Hb (*p* < 0.001), RBCs (*p* < 0.001), and MCV (*p* < 0.001). Similarly, there were linear and quadratic responses in RBC’s (*p* < 0.001 and *p* < 0.001), HCT (*p* < 0.001 and *p* < 0.001), MCH (*p* < 0.001 and *p* < 0.001), platelets (*p* < 0.001 and *p* < 0.001), total leukocyte counts (*p* < 0.001 and *p* < 0.001), and lymphocytes (*p* < 0.001 and *p* < 0.001), as well as quadratic response in Hb (*p* < 0.001), MCV (*p* < 0.001), MCHC (*p* = 0.004) and heterophils (*p* < 0.001)to the *S. frugiperda* meals. 

#### 3.2.2. Serum Bio-Chemistry 

The serum biochemistry of the broiler chickens is summarised in Table 6. The serum biochemistry traits were significant (*p* < 0.001) among the experimental meals. The highest values of creatinine, glucose, cholesterol, and uric acid were determined when broilers were fed the control diet, and the lowest values were registered using the 12% *S. frugiperda* and *H. illucens* meals. The total protein and albumen values were the highest in the 12% *S. frugiperda* diet, while the lowest values were registered using the 4% *H. illucens* meals. The highest and the lowest globulin levels were determined in the broilers fed the 12% *S. frugiperda* and the control meals, respectively. The orthogonal polynomial contrasts showed linear and quadratic responses in glucose (*p* = 0.005 and *p* < 0.001), cholesterol (*p* = 0.002 and *p* < 0.001), total proteins (*p* = 0.002 and *p* = 0.001), globulin (*p* < 0.001 and *p* < 0.001) and uric acid (*p* < 0.001 and *p* = 0.001) while a linear response in creatinine (*p* < 0.001) and albumin (*p* = 0.017) among *H. illucens* meals. Similarly, there were liner and quadratic responses in creatinine (*p* = 0.004 and *p* = 0.041 respectively), glucose (*p* < 0.001 and *p* < 0.001), cholesterol (*p* < 0.001 and *p* < 0.001) and uric acid (*p* = 0.013 and *p* < 0.001); a quadratic response in total protein (*p* < 0.001) and globulin (*p* < 0.001) while a linear response in albumin (*p* = 0.019) among *S. frugiperda* meals. 

### 3.3. Gut Morphometry 

The effects of the meals, intestinal segments, and their interaction with the gut histomorphology indices of the birds are presented in Table 7. There was a significant difference (*p* < 0.001) among meals in terms of Vh, Cd, Vw, and Vh/Cd while sites of the intestine differed significantly (*p* < 0.001) only in Vh and Cd. The interactions between meals and the intestinal sites were significant in Vh, Cd, Vw, and Vh/Cd (*p* < 0.05). The highest Vh value was recorded at the jejunum intestine site of the broilers which were fed the 12% *S. frugiperda* meal, while the lowest values were measured at the jejunum, as well as at the jejunum and ileum sites of broilers fed the control and 4% *H. illucens* meals, respectively. The highest Cd values were found at the jejunum site of the broilers fed the control and the 4% *H. illucens* meals while the lowest Cd values were measured using 12% *H. illucens*, 4% *S. frugiperda*, 8% *S. frugiperda,* and 12% *S. frugiperda* meals at the jejunum and ileum sites. The highest and statistically similar Vw values were recorded using the 12% *H. illucens* meal at the jejunum and the 12% *S. frugiperda* meal at the jejunum and ileum. The lowest and statistically similar Vw values were determined by feeding broilers with the control, 4% *H. illucens* and 8% *H. illucens* meals at the jejunum and ileum sites. The highest and statistically similar ratios of Vh/Cd were recorded in the broilers fed the 12% *H. illucens* and *S. frugiperda* meals at the jejunum and ileum sites as well as the 8% *H. illucens* at the jejunum. Vh/Cd ratios were the lowest using the control meal at the jejunum and ileum sites.

### 3.4. Meat Quality Traits

The meat quality of the broilers fed on different dietary treatments is presented in Table 8. The dietary treatments had a significant difference (*p* < 0.001) on the meat quality traits of broilers except meat pH and shear force (*p* > 0.05). The highest and the lowest cooking loss, drip loss and a* were registered in the meat of broilers fed the control and the 12% *S. frugiperda* diets. The L* was the highest using the 12% *S. frugiperda* diet, while the lowest was determined using the 4% *H. illucens* diet. The highest and the lowest b* values were measured using the 12% *S. frugiperda* and the control meals, respectively. The orthogonal polynomial contrasts showed a linear response in cooking loss (*p* < 0.001) but a quadratic response in L* (*p* < 0.001), a* (*p* = 0.005) and b* (*p* < 0.001) among *H. illucens* meals. Similarly, there were linear and quadratic responses in cooking loss (*p* < 0.001 and *p* = 0.024) and b* (*p* < 0.001 and *p* = 0.04), as well as a linear response in drip loss (*p* = 0.021), L* (*p* < 0.001) and a* (*p* = 0.026) to *S. frugiperda* meals.

## 4. Discussion

In the present study, we assessed how different levels of SBM replacement with *S. frugiperda* and *H. illucens* affect growth performances, haematology, gut morphometry, and meat quality traits in broilers. 

Broiler growth performance is a critical aspect of poultry farming that impacts the overall productivity of the bird. We found variations in the growth performances of broilers fed on different meals of *H. illucens* and *S. frugiperda*. In this study, LW and ADG were higher in broilers fed on 12% *H. illucens* and *S. frugiperda* meals. Insects have higher protein contents and good-quality amino acids than traditional protein sources [48]. It was reported that the larvae of *S. frugiperda* contained 63.5% protein, 22.9% fat, 33.1% lipids, 10.1% fibre, and 5.1% carbohydrates [39,49], while larvae of *H. illucens* comprised 38.2–44.3% protein, 15.1–49% fat, 17–49% lipids, 7.1–10.2% fibre, and 22.15% carbohydrates [50,51,52]. Pieterse et al. [53] and Dabbou et al. [54] noted an increased weight in Cobb 500 and Ross 308 broilers when fed on 15% *H. illucens.* This increase in broiler growth performances could be attributed to the potential use of insects in poultry feed. 

DFI did not show significant differences in response to all the dietary treatments. Feed intake may decrease with increased lipid and protein contents in the insect meals [55,56]. It could also be affected by the feed colour because *H. illucens* meals are darker than those of SBM [57]. Woods et al. [50] and Mat et al. [58] demonstrated that DFI was reduced in broilers and quail birds when fed 10% and 12% *H. illucens*, respectively. The broiler chickens also showed the lowest DFI when fed on 8% *T. molitor* [32] and 30% *M. domestica* meals [59]. Regarding fish meal replacement, 50% of *M. domestica* showed the lowest DFI in Anak broiler chicks [34]. *M. domestica* contains more lysine, threonine, and methionine compared to plant-based sources, and it has an amino acid profile similar to fish meal [59]. Moreover, the apparent digestibility of amino acids in *M. domestica* and fish meals is comparable [60]. Furthermore, *T. molitor* contains more chitin and fat, which may inhibit digestion [61]. Therefore, <5% *T. molitor* inclusion could be administered compared to <50% *M. domestica* [34,62]. Different insect species have varying nutritional compositions; therefore, this could be the reason behind the discrepancy in the findings of the previous studies and the present investigation. 

FCR is one of the essential measures of feed efficiency in birds [63]. In our study, better FCR was recorded in the 12% replacement of SBM with *S. frugiperda* and *H. illucens.* The nutritional profile of insect meals is superior, which could enhance the FCR in broilers [64,65]. Gut development, such as longer Vh and shorter Cd may increase nutrient absorption; therefore, the better FCR observed in this study could be allied with gut development and nutrient absorption [29]. These findings are consistent with those of Schiavone et al. [66], who reported that partial to complete replacement of soybean oil with *H. illucens* improved FCR in Ross 308. According to Ognik et al. [67], young turkeys showed an improvement in FCR when fed with 15% *H. illucens*, whereas Murawska et al. [68] observed that even with 75% *H. illucens*, there was no improvement in FCR in Ross 308. In the case of fishmeal replacement, 100% *H. illucens* showed the lowest FCR in catfish [69].

In the present study, 12% of *H. illucens* and *S. frugiperda* meals resulted in the highest Hb, RBCs, platelets, total leukocyte counts, MCV, heterophil, and lymphocytes. The better nutritional profile of insects, including proteins, saturated fatty acids, and amino acids, may affect the haematological traits in the broilers [52], e.g., palmitic acids stimulate blood production in the boiler [57]. The insect meals improve the immune system by increasing the activity and concentration of birds’ lymphocytes and red blood cells [30]. Our results are aligned with the studies of Biasato et al. [70], who reported a positive impact of insect diet on the haematological traits of broilers (Ross 708). Erythropoiesis (RBC production) in broilers is boosted by protein-rich meals [60,71]. Previous studies suggest that replacing 4 to 100% soybean oil or meal with *H. illucens* has no side effect on the haematological traits in the broiler [67,72,73].

In our study, the lowest levels of creatinine, glucose, cholesterol, and uric acid, as well as the highest levels of total protein, albumen, and globulin have been detected in the broilers fed with the 12% *S. frugiperda* and *H. illucens* meals. Total protein is an important indicator of the physiological state of animals, and it is affected by the quantity and quality of dietary protein [67]. Brede et al. [74], Van Huis et al. [75], and Marono et al. [57] observed that increasing the dietary inclusion levels of *H. illucens* reduced the blood cholesterol and creatinine levels while increasing globulin levels in laying hens. However, some other studies reported that 50 and 100% soybean oil replacement with *H. illucens* had no effect on creatinine, uric acid, and glucose levels in male broiler and female turkeys [76,77]. The chelating effect of chitin in the *H. illucens* could be a possible explanation for this phenomenon [78,79,80]. On the other hand, broilers fed on 0.3% *T. molitor* showed increased levels of serum total protein, albumin, and globulin, while uric acid decreased at 30% inclusion of *T. molitor* [31,41]. The inconsistencies among the results of these studies might be due to differences in poultry breeds, trial conditions, and nutritional profile of insects [55]. 

In the present study, the interactions between meals and the histomorphology of two sites of the intestine were significant. The largest Vh was recorded in the broilers fed the 12% *S. frugiperda* diet at the jejunum site. The Vw and Vh/Cd values were highest at the jejunum and ileum sites using the 12% *H. illucens* and *S. frugiperda* meals, with Cd being the lowest. The structure of the small intestine, i.e., Vh and Cd are widely recognised as the absorptive epithelium for digestion and absorption of nutrients [81]. Larger villi have more surface area for absorbing nutrients, which can lead to efficient nutrient utilisation [82]. The larger Vh and shorter Cd are necessary for proper energy utilisation and digestion, which is important for growth [83,84]. Chu et al. [85] reported that Vh increased while Cd decreased in Hy-line brown chickens at the maximum inclusion level of 9% *H. illucens* in the meals. Our results differ from those of Biaseto et al. [86] and Biaseto et al. [61], who noticed that both jejunum and ileum remained unaffected with meals in terms of gut histological indices, i.e., Vh, Cd, Vw and Vh/Cd in Hubbard hybrid chickens and Ross 708 when fed on 7.5% and 15% *T. molitor*. Dabbou el al. [54] and Dabbou [87] also noticed similar trends in Ross 308 when fed on 15% defatted *H. illucens* and modified fatted *H. illucens* meals. The discrepancies between the results of the current study and the previous studies might be due to differences in breeds, the nutritional value of the insect species and their stages as well as the trial conditions. 

Birds need a healthy gut for efficient digestion and nutrient absorption to acquire the maximum nutrients from meals [88]. The chitin of the insects’ exoskeleton contains antioxidant and antimicrobial peptides that promote food digestion and gut function [89,90]. Proline-rich insect peptides usually comprise 20 to 35 amino acids that improve nutrient digestion and gut health [74]. 

Meat colour is one of the most significant criteria for assessment that influences consumers’ acceptance of meat and its products [91,92]. Colour can be affected by different factors—the pigments of the feed raw materials as well as other ingredients of the formulations—and consumers may interpret the colour based on their preferences [93]. The water-holding capacity of breast meat can be influenced by the high protein content in the diet [94]. In the present study, the 12% *S. frugiperda* showed the minimum cooking, drip losses and a* while the maximum L* and b*. Our findings are comparable with the results of Kim et al. [95], Murawska et al. [68], and Schiavone et al. [96], who observed that increasing the replacement levels of SBM with *H. illucens* decreased the cooking loss while increasing the b* in Ross 308 broilers. Pieterse et al. [97] reported that the water-holding capacity of the meat muscles increases, while thawing and cooking losses decrease in the broiler (Ross 308) when fed on 10% *M. domestica*. Similar trends were observed in quail when fed on 30% *T. milotor* [48]. Leiber et al. [98], Altmann et al. [35], and Pieterse et al. [53] did not observe any effect of *H. illucens* on meat colour in broilers. The meat quality of quail birds did not differ when fed on 15% *H. illucens* [48]. The highest L* can be associated with the oxidation of myoglobin to metmyoglobin, resulting in pale colouration in broiler meat [29]. Moreover, incorporating dietary fat of animal origin into the poultry diet, makes the breast meat lighter L* and more yellow b* than SBM [99]. A study by Kierończyk et al. [100] found that broiler chickens’ L* and b* increased when fed on the diet containing 9% H. illucens meal. However, different factors can affect meat quality, including diet, age, genetics, stress, and slaughtering methods [92].

## 5. Conclusions

The present study showed that the replacement of SBM by 12% of dietary inclusion levels of the larvae of either *S. frugiperda* or *H. illucens* species significantly increased growth performance, haematological and intestinal morphology characteristics as well as improved meat quality. The feeding trials with insect meals increased body weight and average daily weight gain. These meals showed better gut development by replacing SBM with 12% *H. illucens* and *S. frugiperda*, which may enhance the development of the broilers. The observed differences in meat quality with and without substitution of SBM by insect larvae offer important information about dietary interventions that affect meat colour to better suit consumer preferences. Lighter meat is an indication of freshness and tenderness for health-conscious consumers. Therefore, our data highlighted the potential of *S. frugiperda* and *H. illucens* larvae as replacements for SBM in broiler feed for the poultry industry. However, further research is warranted to explore optimal inclusion levels and potential long-term impacts.

## Figures and Tables

**Table 1:** The nutrient compositions, energy contents, and amino acid profiles of *H. illucens* and *S. frugiperda* larvae.

Nutrient Composition ^a^ (%)	*H. illucens*	*S. frugiperda*
Dry matter (as such basis)	91.00	90.00
Crude protein	42.00	47.23
Crude fat	32.60	12.89
Ash	10.70	8.84
Crude fibre	9.40	9.48
Nitrogen-free extract	5.30	21.56
Calcium	2.10	0.37
Available phosphorus	0.94	0.36
**Energy Content (kcal/kg)**	***H. illucens***	***S. frugiperda***
Gross energy	5010	4410
Metabolisable energy ^b^	1464	1284
**Essential Amino Acid (%)**	***H. illucens***	***S. frugiperda***
Arginine	2.26	5.52
Lysine	3.13	6.13
Methionine	1.22	2.03
Threonine	1.88	2.17
Leucine	3.11	6.13
Isoleucine	2.54	3.14
Valine	3.08	3.30
**Dispensable Amino Acid (%)**	***H. illucens***	***S. frugiperda***
Cysteine	0.40	0.72
Tryptophan	0.27	2.20
Glycine	3.06	4.21
Glutamic acid	11.32	14.02
Proline	2.89	2.60
Tyrosine	3.31	3.14
Phenylalanine	4.32	4.20

^a^ Chemical analyses were carried out on three replicates of each feed sample. ^b^ Metabolisable energy was calculated according to Ravindran et al. [6] and all other ingredients were analysed.

**Table 2:** The ingredients, chemical composition, and energy contents of starter meals.

Ingredient (%)	Starter Meal						
	Control	HI4	HI8	HI12	SF4	SF8	SF12
Corn grain	51.17	51.93	54.35	55.57	52.26	53.92	54.35
Wheat bran	4.00	4.00	3.00	3.00	4.00	3.00	3.00
Rice polishing	4.00	4.00	4.00	4.00	4.00	4.00	4.00
Soybean oil	4.00	3.00	1.80	0.80	3.00	2.70	2.50
Soybean meal ^a^	28.50	25.00	21.00	17.00	24.50	20.20	16.00
Fish meal ^a^	6.00	6.00	6.00	6.00	6.00	6.00	6.00
HI meal and SF meals	-	4.00	8.00	12.00	4.00	8.00	12.00
L-Lysine HCl	0.03	-	-	-	-	-	-
DL-Methionine	0.15	0.12	0.10	0.07	0.09	0.03	-
Common salt	0.30	0.30	0.30	0.30	0.30	0.30	0.30
Limestone	1.75	1.55	1.35	1.16	1.75	1.75	1.75
Vitamin premix ^b^	0.05	0.05	0.05	0.05	0.05	0.05	0.05
Micro min premix ^c^	0.05	0.05	0.05	0.05	0.05	0.05	0.05
Total	100	100	100	100	100	100	100
**Nutrient Composition ^a^, (%)**	**Control**	**HI4**	**HI8**	**HI12**	**SF4**	**SF8**	**SF12**
Dry matter	89.40	89.40	89.50	89.40	89.30	89.50	89.40
Crude protein ^d^	23.00	23.03	23.00	22.98	23.03	23.00	22.98
Ether extract	6.65	6.72	6.80	6.86	6.63	6.58	6.59
Crude fibre	4.06	4.22	4.27	4.36	4.22	4.27	4.36
Ash	3.62	3.85	3.96	3.80	3.51	3.63	3.66
NFE ^e^	61.00	60.98	60.84	60.94	61.18	60.91	60.94
Calcium	0.95	0.95	0.95	0.95	0.95	0.95	0.95
Phosphorus (Avail.) ^f^	0.51	0.51	0.52	0.52	0.51	0.52	0.52
Lysine	1.32	1.32	1.33	1.33	1.32	1.33	1.33
Methionine	0.55	0.55	0.55	0.55	0.55	0.55	0.55
Threonine	0.88	0.89	0.90	0.90	0.89	0.90	0.90
Valine	1.03	1.04	1.03	1.04	1.04	1.03	1.04
Arginine	1.41	1.41	1.42	1.40	1.41	1.42	1.40
Leucine	1.46	1.46	1.44	1.45	1.46	1.44	1.45
Isoleucine	0.88	0.89	0.88	0.89	0.89	0.88	0.89
**Energy Content (kcal/kg)**	**Control**	**HI4**	**HI8**	**HI12**	**SF4**	**SF8**	**SF12**
Gross energy	4606	4593	4598	4604	4593	4598	4604
Metabolisable energy ^g^	2980	2978	2973	2974	2978	2973	2973

^a^ Crude protein contents of SBM and fish meal were 45% and 66% (DM basis), respectively. NFE, nitrogen-free extract; ^b^ Starter vitamin premix supplied per kg of diet (formulated and mixed at feed manufacturing site): Vitamin A IU: 12,000, Vitamin D3 IU: 3500, Vitamin E: 30 mg, Vitamin K: 3.0 mg, Vitamin B1: 3.0 mg, Vitamin B2: 8.0 mg, Vitamin B6: 5.0 mg, Vitamin B12: 0.020 mg, Niacin: 40 mg, Pantothenic acid: 18 mg, Folic acid: 2.5 mg, Biotin: 0.24 mg; ^c^ Starter mineral premix: Manganese: 120 mg, Zinc: 100 mg, Iron: 70 mg, Copper: 8.0 mg, Selenium: 0.240 mg, Iodine: 1 mg; Chemical analyses were performed by three replicates of each feed sample; ^d^ Nitrogen-to-protein conversion factor was calculated by Bovera [41] and Janssen [42]; ^e^ NFE, ^f^ phosphorus (avail.) and ^g^ Metabolisable energy were calculated according to Ravindran et al. [6] while all other ingredients were analysed. *H. illucens* (HI).

**Table 3:** The ingredients, chemical composition, and energy contents of grower meals (% as fed).

Ingredient (%)	Grower Meal						
	Control	HI4	HI8	HI12	SF4	SF8	SF12
Corn grain	53.61	55.04	56.97	58.54	55.00	56.56	58.28
Wheat bran	5.00	5.00	4.00	3.00	5.00	4.00	3.00
Rice polishing	4.00	4.00	4.00	4.00	4.00	4.00	4.00
Soybean oil	4.50	3.30	2.30	1.80	3.60	3.20	2.80
Soybean meal	26.00	22.00	18.30	14.50	21.6	17.5	13.2
Fish meal	5.00	5.00	5.00	5.00	5.00	5.00	5.00
HI and SF meals	-	4.00	8.00	12.00	4.00	8.00	12.0
DL-Methionine	0.14	0.11	0.08	0.06	0.08	0.02	-
Common salt	0.30	0.30	0.30	0.30	0.30	0.30	0.30
Limestone	1.35	1.15	0.95	0.70	1.32	1.32	1.32
Vitamin premix ^a^	0.05	0.05	0.05	0.05	0.05	0.05	0.05
Micro min premix ^b^	0.05	0.05	0.05	0.05	0.05	0.05	0.05
Total	100	100	100	100	100	100	100
**Nutrient Composition (%)**	**Control**	**HI4**	**HI8**	**HI12**	**SF4**	**SF8**	**SF12**
Dry matter	89.40	89.30	89.50	89.40	89.30	89.50	89.40
Crude protein	21.51	21.50	21.52	21.50	21.50	21.52	21.50
Ether extract	7.06	7.08	7.10	7.14	7.03	7.02	6.99
Crude fibre	4.06	4.18	4.27	4.31	4.18	4.18	4.21
Ash	3.37	3.55	3.68	3.81	3.39	3.51	3.62
NFE ^c^	62.16	62.20	61.89	61.13	62.20	62.47	62.27
Calcium	0.75	0.75	0.75	0.75	0.75	0.75	0.75
Phosphorus (Avail) ^d^	0.43	0.42	0.42	0.43	0.42	0.42	0.43
Lysine	1.18	1.19	1.19	1.19	1.19	1.19	1.19
Methionine	0.51	0.51	0.51	0.51	0.51	0.51	0.51
Threonine	0.79	0.79	0.79	0.79	0.79	0.79	0.79
Valine	0.91	0.92	0.91	0.92	0.92	0.91	0.92
Arginine	1.27	1.27	1.28	1.27	1.27	1.28	1.27
Leucine	1.30	1.30	1.31	1.30	1.30	1.31	1.30
Isoleucine	0.80	0.81	0.80	0.80	0.81	0.80	0.80
**Energy Content (kcal/kg)**	**Control**	**HI4**	**HI8**	**HI12**	**SF4**	**SF8**	**SF12**
Gross energy	4663	4659	4664	4671	4659	4635	4636
Metabolisable energy ^e^	3055	3049	3048	3051	3049	3048	3051

NFE, nitrogen-free extract; G.E, gross energy; ^a^ Grower vitamin premix supplied per kg of diet (formulated and mixed at feed manufacturing site): Vitamin A IU: 9000, Vitamin D3 IU: 3000, Vitamin E: 25 mg, Vitamin K: 3.0 mg, Vitamin B1: 2.4 mg, Vitamin B2: 6.5 mg, Vitamin B6: 4.2 mg, Vitamin B12: 0.015 mg, Niacin: 35 mg, Pantothenic acid: 15 mg, Folic acid: 1.5 mg, Biotin: 0.21 mg; ^b^ Grower Minerals premix: Manganese: 100 mg, Zinc: 80 mg, Iron: 60 mg, Copper: 8.0 mg, Selenium: 0.240 mg, Iodine: 1 mg; ^c^ NFE, ^d^ phosphorus and ^e^ Metabolisable energy were calculated according to Ravindran et al. [6] while all other ingredients were analysed.

**Table 4:** The ingredients, chemical composition, and energy contents of finisher meals (% as fed).

Ingredient (%)	Finisher Meal						
	Control	HI4	HI8	HI12	SF4	SF8	SF12
Corn grain	57.27	58.65	61.00	63.24	58.77	60.83	62.35
Wheat bran	5.00	5.00	4.00	3.00	5.00	4.00	3.00
Rice polishing	4.00	4.00	4.00	4.00	4.00	4.00	4.00
Soybean oil	5.50	4.30	3.20	2.00	4.80	4.00	3.60
Soybean meal	22.50	18.50	14.50	10.70	17.80	13.60	9.50
Fish meal	4.00	4.00	4.00	4.00	4.00	4.00	4.00
HI and SF meals	-	4.00	8.00	12.00	4.00	8.00	12.00
L-Lysine HCl	0.04	0.03	0.03	-	-	-	-
DL-Methionine	0.14	0.12	0.09	0.06	0.08	0.02	-
Common salt	0.30	0.30	0.30	0.30	0.30	0.30	0.30
Limestone	1.15	1.00	0.78	0.60	1.15	1.15	1.15
Vitamin premix ^a^	0.05	0.05	0.05	0.05	0.05	0.05	0.05
Micro min premix ^b^	0.05	0.05	0.05	0.05	0.05	0.05	0.05
Total	100	100	100	100	100	100	100
**Nutrient Composition (%)**	**Control**	**HI4**	**HI8**	**HI12**	**SF4**	**SF8**	**SF12**
Dry matter	89.40	89.30	89.60	89.50	89.30	89.60	89.50
Crude protein	19.52	19.53	19.51	19.52	19.53	29.51	19.52
Ether extract	8.06	8.08	8.11	8.13	8.01	8.02	7.99
Crude fibre	3.99	4.04	4.08	4.10	3.96	4.08	4.01
Ash	3.44	3.54	3.53	3.57	3.47	3.43	3.51
NFE ^c^	63.82	63.69	63.50	63.40	64.12	64.24	63.96
Calcium	0.65	0.66	0.65	0.66	0.66	0.65	0.66
Phosphorus ^d^ (Avail)	0.36	0.36	0.36	0.36	0.36	0.36	0.36
Lysine	1.08	1.08	1.09	1.08	1.08	1.09	1.08
Methionine	0.48	0.48	0.48	0.48	0.48	0.48	0.48
Threonine	0.72	0.72	0.72	0.72	0.72	0.72	0.72
Valine	0.84	0.84	0.84	0.85	0.84	0.84	0.85
Arginine	1.17	1.18	1.17	1.18	1.18	1.17	1.18
Leucine	1.19	1.19	1.19	1.20	1.19	1.19	1.20
Isoleucine	0.75	0.75	0.76	0.75	0.75	0.76	0.75
**Energy Content (kcal/kg)**	**Control**	**HI4**	**HI8**	**HI12**	**SF4**	**SF8**	**SF12**
Gross energy	4733	4728	4719	4713	4722	4721	4718
Metabolisable energy ^e^	3124	3112	3105	3102	3112	3105	3102

NFE, nitrogen-free extract; G.E, gross energy; ^a^ Finisher Vitamin premix supplied per kg of diet (formulated and mixed at feed manufacturing site): Vitamin A IU: 7000, Vitamin D3 IU: 2500, Vitamin E: 20 mg, Vitamin K: 3.0 mg, Vitamin B1: 1.8 mg, Vitamin B2: 5.0 mg, Vitamin B6: 3.5 mg, Vitamin B12: 0.012 mg, Niacin: 30 mg, Pantothenic acid: 12 mg, Folic acid: 1.0 mg, Biotin: 0.20 mg; ^b^ Finisher Minerals premix: Manganese: 100 mg, Zinc: 80 mg, Iron: 60 mg, Copper: 8.0 mg, Selenium: 0.240 mg, Iodine: 1 mg; ^c^ NFE, ^d^ phosphorus and ^e^ Metabolisable energy were calculated according to Ravindran et al. [6] while all other ingredients were analysed.

**Table 5:** Effect of *H. illucens* and *S. frugiperda* meals on the growth performances of the broiler chickens.

Items	Control	*Hermetia illucens*			*Spodoptera frugiperda*			SEM	*p*-Value				
		HI4	HI8	HI12	SF4	SF8	SF12		ANOVA	HI Lin ^1^	HI Quad ^1^	SF Lin ^1^	SF Quad ^1^
Live weight, g													
DOC	40.05	40.21	40.07	40.09	40.00	40.07	40.12	0.06	0.92	0.99	0.70	0.85	0.60
10 d	230.26 ^e^	247.34 ^d^	263.38 ^c^	292.66 ^ab^	269.70 ^c^	287.17 ^b^	301.00 ^a^	4.23	<0.001	<0.001	0.172	<0.001	<0.001
24 d	1016.97 ^e^	1084.16 ^d^	1147.76 ^c^	1267.82 ^a^	1141.05 ^c^	1166.36 ^c^	1230.56 ^b^	13.72	<0.001	<0.001	0.007	<0.001	<0.001
35 d	1876.62 ^d^	1977.05 ^c^	2050.34 ^b^	2152.38 ^a^	1994.78 ^c^	2052.44 ^b^	2147.45 ^a^	15.16	<0.001	<0.001	0.933	<0.001	<0.001
Average daily gain, g													
1–10 d	23.03 ^e^	24.73 ^de^	26.34 ^cd^	29.27 ^a^	26.97 ^bc^	28.72 ^ab^	30.10 ^a^	0.46	0.01	0.001	0.419	0.112	0.275
11–24 d	56.19 ^e^	59.77 ^d^	63.17 ^c^	69.65 ^a^	62.24 ^c^	62.80 ^c^	66.40 ^b^	0.70	<0.001	<0.001	0.004	<0.001	0.001
25–35 d	78.15 ^c^	81.17 ^ab^	82.05 ^ab^	80.41 ^b^	77.61 ^c^	80.55 ^b^	83.55 ^a^	0.35	<0.001	<0.001	0.073	0.001	<0.001
1–35 d	53.62 ^d^	56.49 ^c^	58.58 ^b^	61.50 ^a^	56.99 ^c^	58.64 ^b^	61.36 ^a^	0.42	<0.001	<0.001	<0.001	<0.001	0.02
Daily feed intake, g													
1–10 d	24.08	24.78	24.17	24.15	24.30	23.94	23.38	0.13	0.168	0.793	0.289	0.377	0.055
11–24 d	92.69	92.94	91.00	92.87	91.53	91.51	91.25	0.32	0.475	0.715	0.353	0.226	0.969
25–35 d	174.75	173.86	173.79	177.24	176.97	176.57	175.73	0.54	0.43	0.319	0.196	0.294	0.38
1–35 d	97.17	97.19	96.32	97.89	97.60	97.34	96.79	0.20	0.49	0.836	0.378	0.134	0.28
Feed conversion ratio, g/g													
1–10 d	1.05 ^a^	1.0 ^a^	0.92 ^b^	0.83 ^d^	0.90 ^bc^	0.84 ^cd^	0.78 ^d^	0.01	0.005	0.002	0.84	0.05	0.84
11–24 d	1.65 ^a^	1.55 ^b^	1.44 ^c^	1.33 ^d^	1.47 ^c^	1.46 ^c^	1.37 ^d^	0.02	<0.001	<0.001	0.66	<0.001	0.03
25–35 d	2.28 ^a^	2.14 ^cd^	2.20 ^bc^	2.11 ^d^	2.24 ^ab^	2.19 ^bc^	2.13 ^d^	0.02	<0.001	<0.001	0.686	0.024	<0.001
1–35 d	1.85 ^a^	1.76 ^b^	1.68 ^c^	1.63 ^d^	1.75 ^b^	1.69 ^c^	1.62 ^d^	0.01	0.003	0.003	0.04	0.045	0.02

DOC, day-old chick; HI4, 4% *H. illucens*; HI8, 8% *H. illucens*; HI12, 12% *H. illucens*; SF4, 4% *S. frugiperda*; SF8, 8% *S. frugiperda*; SF12, 12% *S. frugiperda*; PSEM, Pooled standard error of the mean; ANOVA = Analysis of variance; ^1^ Polynomial contrast; HI lin., *H. illucens* linear; HI quad., *H. illucens* quadratic; SF lin., *S. frugiperda* linear; SF quad., *S. frugiperda* quadratic. The values marked by different letters in superscript within the rows were significantly different (*p* < 0.05).

**Table 6:** Effects of *H. illucens* and *S. frugiperda* dietary treatments on complete blood count and serum biochemistry of the broilers.

Trait	Control	*Hermetia illucens*			*Spodoptera frugiperda*			SEM	*p*-Value				
		HI4	HI8	HI12	SF4	SF8	SF12		ANOVA	HI Lin.	HI Quad.	SF Lin.	SF Quad.
Haematology													
Hb	9.80 ^ab^	8.80 ^c^	9.74 ^ab^	9.80 ^ab^	8.83 ^c^	9.40 ^b^	10.26 ^a^	0.11	<0.001	0.131	<0.001	0.598	<0.001
RBCs	2.92 ^d^	3.49 ^b^	3.21 ^c^	3.60 ^b^	2.91 ^d^	3.12 ^c^	3.92 ^a^	0.06	<0.001	0.228	<0.001	<0.001	<0.001
HCT	31.91 ^b^	28.82 ^f^	30.82 ^d^	31.48 ^c^	28.70 ^f^	29.88 ^e^	33.21 ^a^	0.26	<0.001	0.031	<0.001	<0.001	<0.001
MCV	104.81 ^bc^	88.38 ^d^	89.27 ^d^	108.06 ^b^	83.48 ^e^	103.15 ^c^	116.07 ^a^	1.97	<0.001	0.168	<0.001	0.981	<0.001
MCH	35.76 ^a^	27.75 ^d^	27.91 ^d^	33.96 ^b^	26.85 ^e^	23.95 ^f^	32.09 ^c^	0.68	<0.001	<0.001	<0.001	<0.001	<0.001
MCHC	34.08 ^c^	33.62 ^d^	34.20 ^bc^	35.14 ^a^	34.06 ^c^	33.41 ^e^	34.34 ^b^	0.09	<0.001	<0.001	<0.001	0.221	0.004
Platelets	15055 ^e^	13086 ^f^	15123 ^e^	34961 ^b^	18036 ^d^	19826 ^c^	37937 ^a^	1611.6	<0.001	<0.001	<0.001	<0.001	<0.001
TLC	12120 ^d^	6575 ^g^	11104 ^e^	16218 ^c^	8771 ^f^	18472 ^b^	19125 ^a^	771.07	<0.001	<0.001	<0.001	<0.001	<0.001
Heter.	35.32 ^e^	37.77 ^d^	41.40 ^c^	47.29 ^b^	34.95 ^e^	35.19 ^e^	60.65 ^a^	1.51	<0.001	<0.001	<0.001	0.366	<0.001
Lym.	36.03 ^e^	48.03 ^d^	58.45 ^b^	60.93 ^a^	57.28 ^c^	58.55 ^b^	61.05 ^a^	1.46	<0.001	<0.001	<0.001	<0.001	<0.001
Mono.	1.97	1.95	2.00	2.00	1.97	2.01	1.99	0.03	0.998	0.755	0.905	0.734	0.855
EO.	1.97	1.98	1.97	1.80	1.95	2.00	1.99	0.03	0.637	0.252	0.361	0.576	0.182
Serum biochemistry													
Creatinine	0.46 ^a^	0.42 ^a^	0.38 ^abc^	0.27 ^bc^	0.39 ^ab^	0.36 ^abc^	0.24 ^c^	0.02	<0.001	<0.001	0.073	0.004	0.041
Glucose	212.80 ^a^	201.40 ^ab^	195.80 ^cd^	137 ^f^	198 ^bc^	191.80 ^d^	181.60 ^e^	3.93	<0.001	0.005	<0.001	<0.001	<0.001
Cholesterol	154.20 ^a^	154.40 ^a^	152.20 ^a^	120.40 ^c^	153 ^a^	143.40 ^b^	120.20 ^c^	0.09	<0.001	0.002	<0.001	<0.001	<0.001
T. Protein	3.20 ^ab^	2.78 ^c^	3.23 ^ab^	3.30 ^a^	2.73 ^c^	3.03 ^b^	3.30 ^a^	0.05	<0.001	0.002	0.001	0.15	<0.001
Alb.	1.39 ^c^	1.30 ^c^	1.40 ^bc^	1.48 ^ab^	1.38 ^c^	1.40 ^bc^	1.58 ^a^	0.02	0.027	0.017	0.963	0.019	0.544
Glob.	1.30 ^e^	1.40 ^d^	1.63 ^c^	1.83 ^ab^	1.60 ^c^	1.80 ^b^	1.91 ^a^	0.04	<0.001	<0.001	<0.001	0.814	<0.001
Uric acid	4.30 ^a^	3.41 ^d^	4.08 ^b^	3.61 ^c^	4.17 ^b^	3.50 ^cd^	2.28 ^e^	0.03	<0.001	<0.001	0.001	0.013	<0.001

Traits as follow: Hb g/dL, haemoglobin; RBCs × 10^6^/µL, red blood cells; HCT %, haematocrits; MCV fL, mean corpuscular volume; MCH pg, mean corpuscular haemoglobin; MCHC, mean corpuscular haemoglobin concentration; Platelets/µL; TLC × 10^3^/µL, total leucocytes; Heter. (%), heterophils; Lym. (%), lymphocytes; Mono. (%), monocytes; Eo. (%), Eosinophils; Creatinine mg/dL; Glucose mg/dL; Cholesterol mg/dL; T. protein g/dL, total protein; Alb. g/dL, lbumin; Glob. g/dL, globulin; d Uric Acid g/dL. The values marked by different letters in superscript within the rows were significantly different (*p* < 0.05).

**Table 7:** Influence of *H. illucens* (HI) and *S. frugiperda* (SF) on villus height (Vh), crypt depth (Cd), villus width, and villus height to crypt depth ratio (Vh/Cd) along the intestinal tract of broilers.

Diet	Site	Vh	Cd	Vw	Vh/Cd
Control	Jejunum	1018.0 ^g^	179.21 ^ab^	41.34 ^f^	5.74 ^f^
	Ileum	1113.9 ^f^	166.50 ^bcd^	49.71 ^ef^	6.10 ^f^
HI4	Jejunum	1028.9 ^g^	188.07 ^a^	44.34 ^f^	9.37 ^bc^
	Ileum	1079.2 ^fg^	160.62 ^cde^	48.70 ^ef^	7.57 ^e^
HI8	Jejunum	1407.3 ^de^	168.70 ^bc^	51.474 ^def^	9.86 ^ab^
	Ileum	1387.8 ^e^	149.06 ^ef^	55.76 ^cde^	8.42 ^cde^
HI12	Jejunum	1557.9 ^b^	155.15 ^cdef^	72.06 ^ab^	10.16 ^ab^
	Ileum	1477.0 ^cd^	145.88 ^f^	63.47 ^bc^	9.46 ^abc^
SF4	Jejunum	1432.1 ^de^	153.82 ^def^	59.118 ^cde^	9.08 ^bcd^
	Ileum	1123.6 ^f^	148.56 ^ef^	59.86 ^cde^	7.63 ^e^
SF8	Jejunum	1482.1 ^cd^	153.77 ^def^	61.42 ^bcd^	9.32 ^bc^
	Ileum	1435.0 ^de^	148.29 ^ef^	63.89 ^bc^	7.79 ^de^
SF12	Jejunum	1759.9 ^a^	145.08 ^f^	80.17 ^a^	10.76 ^a^
	Ileum	1511.8 ^bc^	142.81 ^f^	76.88 ^a^	9.93 ^ab^
PSEM		15.89	2.95	2.39	0.34
	Probability				
Diet		<0.001	<0.001	<0.001	<0.001
Site		<0.001	<0.001	0.35	0.33
Diet * Site		<0.001	<0.001	0.01	<0.001

Seven experimental meals were tested: Control; HI4, 4% *H. illucens*; HI8, 8% *H. illucens*; HI12, 12% *H. illucens*; SF4, 4% *S. frugiperda*; SF8, 8% *S. frugiperda*; SF12, 12% *S. frugiperda*. Two intestinal sites, jejunum, and ileum. PSEM, Pooled standard error of mean. * Effects of two variables have been combined in ANOVA analyses. The values marked by different letters in superscript within the columns were significantly different (*p* < 0.05).

**Table 8:** Effects on the meat quality of broilers fed on *H. illucens* and *S. frugiperda* meals.

Trait	Control	*Hermetia illucens*			*Spodoptera frugiperda*			SEM	*p*-Value				
		HI4	HI8	HI12	SF4	SF8	SF12		ANOVA	HI Lin.	HI Quad.	SF Lin.	SF Quad.
Cooking loss	33.81 ^a^	28.55 ^b^	26.67 ^bc^	22.79 ^de^	28.61 ^b^	24.88 ^cd^	20.10 ^e^	0.80	<0.001	<0.001	0.45	<0.001	0.024
Drip loss	2.69 ^a^	2.24 ^ab^	2.18 ^abc^	1.98 ^bc^	1.54 ^cd^	2.59 ^ab^	1.19 ^d^	0.12	0.003	883	0.072	0.021	0.315
Meat pH	6.13	6.13	6.16	6.17	6.13	6.18	6.14	0.01	0.896	0.304	0.957	0.578	0.894
Shear Force	61.40	62.61	61.64	60.76	61.42	60.59	60.57	0.22	0.133	0.27	0.082	0.26	0.297
L*	50.15 ^c^	48.98 ^c^	54.83 ^b^	57.68 ^ab^	55.88 ^b^	56.93 ^ab^	59.10 ^a^	0.70	<0.001	0.251	<0.001	<0.001	0.795
a*	15.68 ^a^	15.57 ^a^	15.62 ^ab^	14.17 ^bc^	14.70 ^ab^	14.09 ^bc^	12.94 ^c^	0.22	<0.001	0.12	0.005	0.026	0.676
b*	13.40 ^d^	15.39 ^c^	15.56 ^c^	17.72 ^b^	15.32 ^c^	16.50 ^bc^	20.38 ^a^	0.41	<0.001	0.06	<0.001	<0.001	0.04

L*, Lightness; a*, redness; b*, yellowness; HI4, 4% *H. illucens*; HI8, 8% *H. illucens*; HI12, 12% *H. illucens*; SF4, 4% *S. frugiperda*; SF8, 8% *S. frugiperda*; SF12, 12% *S. frugiperda*; PSEM, Pooled Standard Error of Mean. The values marked by different letters in superscript within the rows were significantly different (*p* < 0.05).

## Data Availability

The original contributions presented in the study are included in the article. Further inquiries can be directed to the corresponding authors.
